# From Powder to Pathogen: A Systematic Review of Detection, Pathogenicity, and Mitigation of *Cronobacter sakazakii* in Infant Formula

**DOI:** 10.1155/ijm/7658756

**Published:** 2026-06-07

**Authors:** Sutapa Bhowmik, Sangita Ahmed, Fatema Hasan Kaifa, Md. Abdullah Al Safi, Supantha Rivu, Md. Latiful Bari, Md. Aminul Islam, Nayan Chandra Mohanto

**Affiliations:** ^1^ Department of Microbiology, Noakhali Science and Technology University, Noakhali, Bangladesh, nstu.edu.bd; ^2^ Department of Microbiology, University of Dhaka, Dhaka, Bangladesh, du.ac.bd; ^3^ Department of Microbiology, Notre Dame University Bangladesh, Dhaka, Bangladesh; ^4^ Food, Nutrition and Agriculture Research Division, Centre for Advanced Research in Sciences, University of Dhaka, Dhaka, Bangladesh, du.ac.bd; ^5^ Department of Biochemistry and Molecular Biology, Shahjalal University of Science and Technology, Sylhet, Bangladesh, sust.edu

**Keywords:** antimicrobial resistance, biofilm, *Cronobacter sakazakii*, meningitis, opportunistic pathogen, powdered infant formula

## Abstract

*Cronobacter sakazakii* is an emerging foodborne pathogen that has gained increasing global attention due to its association with severe infections in neonates, particularly meningitis, sepsis, and necrotizing enterocolitis. These infections are often associated with contaminated powdered infant formula (PIF), a nonsterile but commonly used alternative to breast milk. We conducted a systematic review using the PRISMA approach and registered it in PROSPERO to synthesize current scientific knowledge on the detection, pathogenic mechanisms, antimicrobial resistance, and mitigation strategies *of C. sakazakii*. A total of 82 research articles published between 2009 and 2024 were included. The findings highlight the bacterium′s ability to survive under harsh environmental conditions, including desiccation, osmotic stress, and heat, making it a persistent contaminant in food production. Virulence is driven by several genetic determinants, including outer membrane proteins (*OmpA* and *OmpX*), invasion‐associated genes (*InvA* and *cpa*), and iron acquisition systems. Sequence types ST4 and ST1 are consistently linked to neonatal infections and show strong biofilm‐forming potential, increasing the risk of recurring contamination in manufacturing environments. The review compares conventional culture‐based detection methods with advanced molecular techniques such as polymerase chain reaction (PCR), multilocus sequence typing (MLST), and aptamer‐based biosensors, highlighting the need for rapid, cost‐effective, and accurate diagnostic tools. While most strains retain antibiotic susceptibility, emerging resistance to *β*‐lactams, macrolides, and cephalothin has been reported, varying by geographic region and strain type. The LED‐based decontamination, citral treatments, and antimicrobial packaging materials are promising avenues for prevention. Furthermore, this review underscores the importance of enhanced regulatory oversight, standardized protocols, and public awareness regarding the safe preparation and handling of infant formula, considering the vulnerability of infants. Continued interdisciplinary research and international collaboration are warranted, ensuring microbiological safety and protecting infant health.

## 1. Introduction

Following the 2001 meningitis outbreak in Tennessee, United States, a motile peritrichous nonspore forming facultative anaerobic gram‐negative rod came to the attention of the world as a newly discovered opportunistic pathogen [1, 2]. Following its reclassification in 2007, this pathogen, which was previously known as *Enterobacter sakazakii* in the taxonomic classification, is now known as *Cronobacter sakazakii*. This reclassification split the original species into seven different species within the *Cronobacter* genus, including *C. sakazakii*, *C. malonaticus*, *C. turicensis*, *C. muytjensii*, *C. dublinensis*, *C. universalis*, *and C. condimenti* [3, 4] Three of the seven species in this genus *Cronobacter*, which includes *C. sakazakii*, *C. turicensis*, and *C. malonaticus*, are linked to severe and systemic infections in infants under 12 months, particularly neonates [1]. Contaminated powdered infant formula (PIF) has been linked to these organisms and ultimately to the infections are reported over the years, which may lead to meningitis, sepsis, or necrotizing enterocolitis [1, 5–8]. There are regional variations in the prevalence of *C. sakazakii* in PIF, with ~38% in North America followed by ~18% in South America, ~13% in Africa (13.00%), ~8% in Asia, and ~5% in Europe [9]. Approximately 40% of newborns diagnosed with bloodstream infections and meningitis caused by *Cronobacter* spp. have resulted in deaths worldwide. The Centers for Disease Control and Prevention (CDC) received 76 reports of severe newborn *Cronobacter* infections in the United States between January 2002 and July 2018 [10]. A nationwide assessment of 3600 PIF samples found an overall contamination rate of 0.75%, with the highest rate of 1.25% reported in Shaanxi province in China [10]. The occurrence of infection raises concerns about the need for a comprehensive understanding of its transmission, resistance, control, and prevention.

Compared with other *Cronobacter* species, *C. sakazakii* strains have the unique ability to adapt to a vast range of extreme environments. Strains of ST4, ST1 [7], and ST83 [11] can withstand acid stress, heat, desiccation, and osmotic stress [9, 12]. Premature delivery and/or low birth weight children are among the high‐risk categories; nevertheless, their lack of an established gut epithelial lining and normal gut bacteria leave them more vulnerable to an increased mucosal permeability [13]. According to reports, newborn meningitis caused by *C. sakazakii* has a fatality rate of 50%, with half of the affected infants dying within a week of diagnosis [14]. Despite the emergence of resistant strains, most of the strains of *C. sakazakii* are still generally susceptible to a number of antibiotics. However, strains such as ST4, ST1, ST83, ST64, and ST148 are found to be resistant against certain antibiotic groups such as sulfonamides, fluoroquinolones, carbapenems, and beta lactams [15–17]. Alternative control techniques such as light‐emitting diode (LED) exposure [18, 19] combined citral and LED treatments, and antimicrobial AgNP‐based films [20] have demonstrated encouraging efficacy in decreasing its viability. The pathogen is extremely dangerous for neonates′ health, infecting them severely and fatally, especially in preterm or immunocompromised babies. Notwithstanding the progress made in detection and prevention, the nonsterile nature of powdered formula still poses challenges [17]. Infants can also be protected from exposure if caregivers follow proper formula preparation and storage guidelines. There is no gold standard detection method for clinically identifying this threatening organism. Accordingly, researchers and microbiologists have to rely on traditional culture–based methods and biochemical analysis to detect *Cronobacter* spp. [21]. Additionally, the mechanism of multidrug resistance is yet to be clarified, and alternative approaches to treating this infection must be considered subsequently.

This study provides a comprehensive analysis of the microbiological and molecular characteristics of *C. sakazakii*, with a particular focus on its relevance in PIF. It explores the pathogen′s pathophysiology, survival mechanisms, and immune evasion strategies that contribute to its clinical impact in neonates. Diagnostic approaches are evaluated by comparing conventional microbiological techniques with advanced molecular methods to identify the most efficient detection strategies. The study also investigates the antimicrobial resistance (AMR) profiles of isolated strains, highlighting treatment challenges posed by emerging resistance. In addition, it assesses existing mitigation measures across the PIF production chain, from manufacturing and storage to handling, examining epidemiological trends, risk factors, and the effectiveness of current regulatory frameworks ensuring infant formula safety.

## 2. Materials and Methods

### 2.1. Search Strategy and Study Selection

A search strategy was designed by strictly adhering to PRISMA recommendations to comprehensively identify all relevant original research articles focused on the possible diseases caused by *C. sakazakii* isolates from PIF ingestion. We searched three electronic databases (PubMed, Embase, and Scopus) independently to ensure the validity and minimize the selection bias. The timeframe of this study was restricted to the period of January 2009 to December 2024 to reflect the most recent advancements in the field. The screening for study selection and data extraction was performed by three independent coauthors. Screening of titles and abstracts was done for relevance, followed by a complete text review as per predefined inclusion and exclusion criteria.

**Figure 1:**
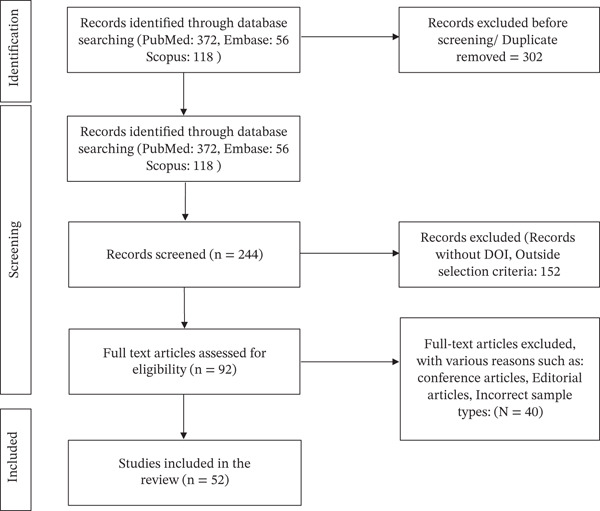
Flowchart showing identification, screening, and inclusion and exclusion of the original research articles to the current review study.

At first, the articles with selected keywords “*Cronobacter sakazakii*” AND “infant” and “*Cronobacter sakazakii*” AND “powdered infant formula” in PubMed, “*Cronobacter sakazakii*” AND “infant formula” in Embase, and “*Cronobacter sakazakii*, infant, powdered infant formula” in Scopus were screened out. After the initial screening, 384 journal articles were retrieved for secondary screening. Later, 276 duplicates of the articles from three databases and because of the unavailability of full texts were excluded. Another 26 articles were excluded as they were conference papers, editorial articles, or the samples were found to be different from PIF and RTE food. Finally, 82 journal articles were included in the review (Figure 1).

### 2.2. Exclusion and Inclusion Criteria and Screening of Relevant Studies

#### 2.2.1. Exclusion Criteria

All qualitative research was excluded, such as studies employing narrative analysis or other nonnumerical approaches, as these do not align with the empirical quantitative focus of the study. Additionally, secondary literature, including review studies, systematic reviews, meta‐analyses, narrative reviews, and umbrella reviews, was rejected to emphasize original research findings. Although the primary focus of our study was on the relationship between PIF and *C. sakazakii* in human populations, studies based on animal models or involving nonhuman subjects were also considered. Opinion‐based literature, including commentaries and editorials, was also excluded to ensure that the review is grounded in scientific evidence rather than subjective perspectives or anecdotal claims. Furthermore, studies published in languages other than English were not taken into consideration to avoid inconsistent interpretation. The duplicates were removed; the titles and abstracts were reviewed to exclude articles that did not meet the predefined inclusion criteria.

#### 2.2.2. Inclusion Criteria

This study was focused on empirical primary quantitative studies that provide robust evidence by reporting statistical relationships between infant formula and *C. sakazakii*. This study would ensure a data‐driven understanding of the association and highlight significant findings relevant to the subject. To capture a comprehensive range of perspectives and methodologies, eligible studies encompassed a variety of observational designs, including prospective and retrospective cohort studies, case reports, case series, case‐control studies, and cross‐sectional studies, allowing for a nuanced exploration of both individual cases and population‐level trends. To maintain linguistic consistency and accessibility for global researchers, only studies published in the English language were included, avoiding potential language‐related misinterpretations. Furthermore, to reflect the latest advancements and current scientific understanding, the review was exclusively considered research published within the last 15 years, ensuring that the findings are up‐to‐date and relevant to modern contexts. The targeted database search terms were carefully chosen to capture the critical intersection between the pathogen, its primary transmission vehicle, and the vulnerable infant population. Subsequently, the full texts of the remaining studies were meticulously examined by considering the inclusion and exclusion criteria. This step involved assessing each article for its study design, relevance to the topic, statistical analysis of the association between *C. sakazakii* and PIF, and adherence to the 15‐year publication window. Each full‐text screening was conducted independently by three coauthors to enhance reliability and minimize selection bias. Any discrepancies in the selection process were resolved through discussion or consultation to ensure accuracy and consensus in the final selection of articles.

### 2.3. Data Extraction

The procedure encompassed the screening of text, followed by the examination of tables and graphs. To establish confidence in the extracted data, it was tested in triplicate. The extracted data included details such as the name of the first author, year of publication, the bacterial species and strains, different methods for their protein and DNA analysis, places of outbreaks and prevalence, method of detecting biofilm formation, and methods of detecting diseases caused, possible treatments and advancement, different environmental factors responsible for their survival in PIF milk and making their treatment difficult, and biochemical analysis of the bacteria. After collecting and analyzing all the information, this record for the systematic review was registered to the Prospective Register of Systematic Reviews (PROSPERO) under the title “A Multifaceted Review of *Cronobacter sakazakii*: An Emerging Foodborne Pathogen in Powdered Infant Formula”. The registration ID is CRD42023494149.

## 3. Results and Discussion

### 3.1. Microbiology of *C. sakazakii*

Modified lauryl sulfate tryptose broth‐vancomycin medium (mLST‐Vm), from Merck (Germany) and Thermo Fisher/Oxoid (United States/United Kingdom) are the most used globally for enrichment of facultative anaerobic, gram‐negative, nonspore‐forming bacteria like *C. sakazakii* [5, 22] In particular, for selective isolation of *C. sakazakii*, chromogenic *E. sakazakii* isolation agar—ESIA (CF 3706, Biolife, Italy) [23, 24], Brilliance Chromogenic Agar CM 1035 (Oxoid Thermo Fisher, United Kingdom) [25], Druggan–Forsythe–Iversen agar (DFI, Beijing Obostar Biotechnology Co. Ltd., China) [5], and CHROMagar *E. sakazakii* (DRG International) [22, 26] are some of the selective media that are readily accessible for the cultivation of these bacteria. In DFI medium, *C. sakazakii* often produces blue‐green colonies, and in ESIA medium, the organism produces green colonies, whereas the newer CM or CHROMagar media yield blue‐green/turquois colonies. The bacteria′s complete genome sequence size ranges from 4.4 to 4.7 Mb. More than 98% of the genome is made up of coding sequences (CDSs) and a GC content that varies from 56.35 to 57.06% [7, 27–29].

#### 3.1.1. Sequence Typing of *C. sakazakii*

**Figure 2:**
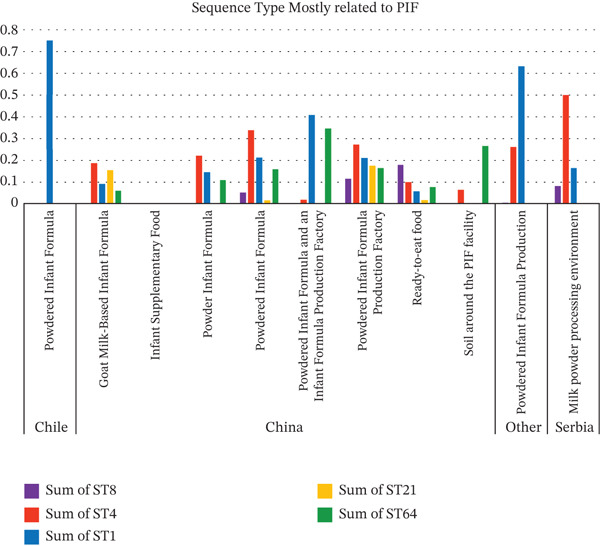
Graphical presentation of the sequence types mostly associated with powdered infant formula (PIF).

For *Cronobacter* spp., the seven housekeeping genes are *atpD*, *fusA*, *glnS*, *gltB*, *gyrB*, *infB*, *and ppsA*, which are vital for bacterial life and are typically safeguarded among strains and are used to determine the sequence types (STs) by standard multilocus sequence typing (MLST) analysis [30]. Eleven studies explored the STs of this pathogen using MLST, and the most common STs reported from PIF were ST4, ST1, ST8, ST64, and ST21 (Table 1, Figure 2). A relationship map has been prepared to identify the STs isolated directly from PIF sources (Figure 2). In a thorough investigation, Fei et al. (2022) examined 3600 commercial PIF samples that were gathered from nine Chinese regions between March 2018 and September 2020 [10]. They isolated 27 *Cronobacter* isolates, including 22 *C. sakazakii*, and they discovered 14 STs, with ST1 and ST4 being two of the most common sequences. The study also evaluated the isolates′ capacity to form biofilms, observing that several STs specifically, ST1 and ST4 showed potent biofilm‐forming capacities, which would explain why they persisted in PIF production settings [10]. Clinical isolates of *C. sakazakii* linked to newborn meningitis in the United States were the main focus of Hariri et al. (2013) [37]. All five of the CSF isolates they looked at were found to be members of ST4 or its clonal complex [37]. Therefore, all these studies suggest that ST4 is the most commonly found ST that is involved with neonatal meningitis.

**Table 1:** Sequence types mostly associated with PIF.

Row labels	Sum of ST8	Sum of ST4	Sum of ST1	Sum of ST21	Sum of ST64	Ref.
Chile	**0**	**0**	**0.75**	**0**	**0**	[31, 32]
Powdered infant formula	0	0	0.75	0	0	
China	**0.35215**	**1.2085**	**1.1362**	**0.37113**	**1.19524**	[33–36]
Goat milk–based infant formula	0	0.1875	0.0938	0.1563	0.0625	
Infant supplementary food	—	—	—	—	—	—
Powder infant formula	0.0009	0.2222	0.1481	0.000455	0.1111	
Powdered infant formula	0.0536	0.339	0.214	0.0179	0.161	
Powdered infant formula and an infant formula production factory	0	0.02041	0.40816	0	0.34694	
Powdered infant formula production factory	0.11765	0.2727	0.2121	0.1765	0.1667	
Ready‐to‐eat food	0.18	0.1	0.06	0.02	0.08	
Soil around the PIF facility	0	0.06667	0	0	0.267	
Other countries in Europe	**0.008**	**0.263**	**0.632**	**0**	**0**	
Powdered infant formula production	0.008	0.263	0.632	0	0	
Serbia	**0.0833**	**0.5**	**0.1667**	**0**	**0**	[17]
Milk powder processing environment	0.0833	0.5	0.1667	0	0	
Grand total	**0.443447059**	**1.97147483**	**2.684863265**	**0.371125134**	**1.195238776**	

*Note:* The country‐wise and the grand total data are marked by bold.

### 3.2. Risk Factors

*C. sakazakii*, a pathogen primarily found in dehydrated foods with low water activity, poses a significant risk, particularly those in PIF. Based on the age and health status of individuals, its impact varies; adults may show few or no symptoms, but in infants under 12 months and immunocompromised individuals (though rare), including the elderly, cancer patients, those with AIDS, and recipients of organ transplants, the infection can be devastating [38, 39]. The severe consequences include sepsis, meningitis, necrotizing enterocolitis, and tragically, death [38]. The bacterium is especially concerning for premature infants with low birth weights and those suffering from malnutrition. In severe cases, brain abscesses, seizures, and elevated leukocyte counts may also result, potentially leading to enduring brain issues related to sensory development [25]. The brain developmental disease is mostly found among premature infants with lower birth weights and those babies that are having malnutrition, such as babies with low iron levels [38]. The United States Food and Drug Administration (US FDA) has issued warnings regarding the presence of *C. sakazakii* in PIF and recommends special care in preparing formula for infants who are less than 2 months old, born prematurely, or babies who have a weakened immune system [40]. Although the primary focus has been on PIF due to its direct link to several cases of *C. sakazakii* infection, RTE (ready‐to‐eat) foods for infants can also be a potential source of this pathogen. An international survey reported the presence of *C. sakazakii* at least in 27 different food products among the total of 290 products examined [41]. The bacterium′s ability to survive in low‐moisture foods makes it a concern for any RTE products that may not undergo thorough cooking or sterilization before consumption, for example, sandwiches, salad, bakery items, sushi, oysters, and shellfishes [5].

### 3.3. Sources

In 2024, Lindsay et al. emphasized that PIF was recognized as one of the potential sources of *C. sakazakii* because of its capability to survive in harsh environmental conditions [42]. The bacterium is capable of surviving in the dry environment of PIF for extended periods [42]. Since PIF is not manufactured as a sterile product, there is a risk of contamination. The bacterium can enter manufacturing facilities on hands, shoes, and other contaminated surfaces [43]. The potential of *C. sakazakii* to create biofilms, which are organized communities of bacteria covered in an extracellular matrix that the bacteria produce on their own, is a key component of their persistence. These biofilms are resistant to typical cleaning and disinfection techniques because they can stick to a range of surfaces that are frequently seen in manufacturing settings, such as silicone, latex, and stainless steel [44]. Therefore, these investigations have revealed insanitary conditions within facilities, including the presence of different strains of *C. sakazakii*, which can cause contamination of the formula. However, Cechin et al. (2023) point out that *C. sakazakii* is present in a variety of meals derived from plants and is not just prevalent in dairy products [45]. Exposure to polluted soil, compost, animal waste, and agricultural water are examples of contamination pathways. Its presence in plant‐based food products can also be facilitated by the bacterium′s ability to persist in household and processing environments [45]. Conclusively, contamination with *C. sakazakii* can come from a variety of sources, such as plant‐based foods, powdered baby formula, and environmental reservoirs.

### 3.4. Routes of Contamination

*C. sakazakii* is frequently associated with PIF, particularly when reconstituted with water. Due to the heat sensitivity of micronutrients in PIF, they go through postheat treatment to preserve nutritional content, in accordance with requirements. Infant formula, either in powdered, liquid concentrate, or ready‐to‐feed forms, is the sole appropriate substitute for newborns unable to breastfeed, as cow′s milk is deficient in vital nutrients [39]. Transmission of *C. sakazakii* occurs via three primary pathways: (i) PIF, wherein the bacterium endures in dry environments and contaminates during production or reconstitution [40]; (ii) environmental sources, including contaminated surfaces in domestic, medical, or food processing settings [43]; and (iii) contaminated feeding apparatus, such as breast pumps, highlighting the necessity for adequate sterilization [43]. The production of a safe formula is complex due to the necessity of nutritional additions and sterilization processes. Moreover, *C. sakazakii* might infect newborns due to inadequate sanitation during food preparation or from contaminated feeding utensils. At‐risk populations, such as premature or immunocompromised newborns in healthcare and childcare settings, encounter increased vulnerability [10]. Breastfed children may contract infections through cross‐contaminated expressed milk from improperly sanitized pumps [25, 43].

### 3.5. Pathogenesis of *C. sakazakii*

#### 3.5.1. Virulence

The approach by which *C. sakazakii* causes disease is aided by multiple virulence genes. There is evidence that the virulence plasmid carries the *cpa* and *iucABCD* (*iucA*, *iucB*, *iucC*, *iucD*) genes [46]. However, an exception is noticed in another virulent strain, ST8 that carries the pESA3‐like virulence plasmid, but lacks *cpa* gene [47, 48]. Numerous genes and proteins allow bacteria to withstand extreme conditions such as elevated temperatures, osmotic stress, desiccation, and more. Genes associated with the regulation of capsular polysaccharides (CPS) are linked to both osmotic stressors and desiccation tolerance [24].

Other genes found in this bacterium include *bcsC*, *bcsG*, *fliD*, *flgJ*, *bcsA*, and *flhE*, which have been linked to the development of biofilms [49]. Silicon, stainless steel, polycarbonate, and latex were all suitable surfaces for *Cronobacter* spp. to cling to; bacteria that produced extracellular polysaccharides, or EPS, appeared to have a stronger affinity for these materials. It has been determined that colanic acid (CA) is a component of EPS in *Cronobacter* spp. that increases tolerance to environmental stressors such as heat, desiccation, and pH and promotes adhesion to a variety of surfaces [50]. The genes linked to their survival traits are summarized in Table 2.

**Table 2:** Genes responsible for bacterial survival in the sample/host body.

Genes/proteins	Essential for	Reference
degP	Function as the periplasmic serine endo protease needed for cell existence at high temperatures.	[51]
ibpA, ibpB	Heat shock proteins	[52, 53]
hsIT	Escalates membrane permeability, which could subsequently extend the longevity of cells in challenging environments	[51, 54]
Clp protease	ATP‐dependent as well as able of breaking down or folding broken proteins in bacterial cells when they are experiencing stress, such as at elevated temperatures	[55]
cspC and cspE	Cold shock proteins	[55]
speA	Gene that codes for ribosomal proteins that shields bacteria against environmental stresses such as extreme salinity, osmotic stress, reactive oxygen species, and acidic conditions.	[56]
Chaperone proteins—clpB, dnaK, hfq, surA and dnaJ	Assists in rendering cells more resistant to desiccation and harsh situations.	[52, 57]
TrkA	Encodes a mechanism for absorbing potassium and is necessary to withstand desiccation along with osmotic conditions.	[58, 59]
LeuO	Ubiquitous transcriptional activator that influences several biological functions, including as the reaction to stressful circumstances	[60]
envZ	Affects bacterial resistance to biofilm formation, desiccation as well as flagella movement. It also encodes the sensory histidine kinase for osmolarity.	[60, 61]
LPSs	Heat‐tolerant	[62]
TerC	Membrane protein involved in tellurium resistance	[63]
ProP	Osmolyte uptake system	[64]
Cyx	Regulator of the envelope distress to enhance the integrity of the cell envelope	[64]
RpoS	The desiccation persistence of *C. sakazakii* is dependent on the stress response sigma factor, which is crucial in controlling the species′ reaction to osmotic stress.	[52, 56]
OmpL (outer membrane porin L)	A gene responsible for *C. sakazakii*′s sensitivity to heat stress.	[52]
thrB‐Q genes	Longer version of thermotolerance islands (18 kbp)	[52]
thrBCD and thrOP genes	Shorter version of thermotolerance islands (6 kbp)	[52]
RimP (ribosome maturation protein)	Plays an indispensable role in ribosome assembly at extreme temperatures.	[52]
orfI	A DNA marker for thermotolerance that is tightly associated with *C. sakazakii*′s enhanced thermoresistance.	[52]
infB	Component that initiates translation and contributes to heat resistance.	[52]
CRISPR‐Cas systems (I‐E and I‐F)	These mechanisms contribute to the defense against genetic elements that are mobile such as phages.	[65–67]
hcp (Hcp)	This gene encodes the receptor/chaperone Hcp and is a component of the Type VI secretion system (T6SS). It contributes to both host interaction along with bacterial competitiveness.	[65, 66]
iutA	Through its binding to the iron‐scavenging siderophore aerobactin, the ferric aerobactin receptor IutA participates in the acquisition of iron.	[65, 66]
entB	Another siderophore associated with iron absorption, enterobactin production, depends on enterobactin synthase component B (EntB).	[65, 66]
Fep	For enterobactin to penetrate the outer membrane, the ferric enterobactin outer membrane transporter (Fep) is indispensable.	[65–67]
Fes	Within the bacterial cell, enterobactin is processed by the ferric enterobactin esterase (Fes).	[65–67]
Clvp	The survival and fitness of bacteria are correlated with the ATP‐dependent protease ClpV.	[65, 66, 68]
cpsG	Phosphomannomutase CpsG is involved in the manufacture of colanic acid, which is necessary for the creation of biofilms and pathogenicity.	[65, 66]
cdiA	In interbacterial competition, the contact‐dependent inhibition effector toxin CdiA serves an integral part.	[65, 66]
Hec	Associated to B family hemolysin secretion/activation.	[65–67]
icmF1/tssM1/hsiC1/vipB/tssC	The Type VI secretion system (T6SS), which is linked to this gene, is involved in host contact with bacterial competitiveness.	[65]
iucA	Aerobactin synthase, which is produced by this gene, aids the bacteria in scavenging iron from the host environment.	[65–67]
ompA	Bacterial survival alongside persistence is aided in the production of biofilms by the outer membrane protein A (OmpA).	[65, 66]
kpsD	To help in immune evasion, the capsule polysaccharide ABC transporter substrate‐binding protein (KpsD) is necessary for capsule production.	[65–67]

The invasion protein InvA and the outer membrane proteins OmpA, OmpX, and OmpF are essential components of *C. sakazakii*′s pathogenicity. These membrane‐associated proteins are expressed at higher levels in virulent strains of *C. sakazakii* than in less virulent strains. Higher levels of adhesion, invasion, and survival in host settings are correlated with this differential expression [27]. The bacteria finish their pathogenic mechanisms by effectively entering the host cell and then start residing there. The persistence strategy comes after acquisition of iron, utilization of sialic acid, and resistance to immune system invasion. The invasion framework includes translocation, quorum sensing, secretion system, motility, colonization, and resistance to antimicrobial therapeutics (AMR) [69].

#### 3.5.2. Cell Adhesion and Invasion Mechanism

Bacteria adhere to host cells in diverse circumstances. The permeability of the neonatal gut wall increases, and epithelial tight junctions become weaker by *manB*, *kdsA*, the lipopolysaccharide (LPS)‐encoding genes *VC0243*, and *gtrA* [50]. Flagellar actions along with the chemotaxis lead to bacterial cell attachment, which later allows the bacteria to infiltrate into the cells. The interaction between genes linked to chemotaxis *cheY* and *motA* and flagella‐associated genes, *flhA*, *fimB*, *flgB*, *flhC*, *flgG*, *fliG*, *flgH*, *fliM*, *fliQ*, *and flip* causes adhesion and subsequently invasion [7]. In *C. sakazakii*, the flagellar hook–associated protein FlgK is essential for cell adhesion and invasion, as well as desiccation resistance. Mutations in the *flgK* gene result in reduced motility and adhesion capabilities, highlighting its importance in virulence [20]. Fimbriae, particularly curli fimbriae, are thin, coiled, highly aggregative amyloid fibers that provide an amorphous matrix facilitating adhesion. They are involved in rugosity, cell aggregation, and adhesion to surfaces, contributing to biofilm formation and persistence in various environments [44]. Furthermore, binding with the receptors expressed on the apical and basolateral host gut cell membrane surfaces, the *ompA* and *ompX* genes in *C. sakazakii* possess vital functions for both adherence and invasion [49, 50, 70–73]. Additionally, it has been revealed that *OmpX* facilitates the migration of *C. sakazakii* into deeper organs of rats, such as the liver and spleen. By inactivating the plasmin inhibitor *α*2‐AP and activating plasminogen, the plasmid‐encoded outer membrane protease Cpa may facilitate widespread bacterial invasion of human tissues and provide resistance to serum [70]. It has been descried that the *ibeB* homolog encoded by the *cusC* gene is connected to the invading human brain microvascular endothelial cells [5], [72]. This gene may contribute to the emergence of newborn meningitis, as evidenced by the two clinical instances that have been documented in Brazil and China [50], [73]. Furthermore, *OmpA* has been implicated in *C. sakazakii* invasions of intestinal cells, blood‐brain barrier, central nervous system (CNS), and epithelial cells, all of which hold significance for pathogenicity and clinical symptoms [7]. According to one publication, *C. sakazakii* mutants missing the RNA‐binding protein *hfq* exhibit to have defects in their ability to invade mammalian cells and survive there [49]. The *NanAKT* gene cassette enables *C. sakazakii* to use sialic acid, essential for host neurological development, hence promoting extensive infection establishment. [74]. Several possible *Cronobacter* virulence‐related components encompass zinc‐containing metalloprotease (*zpx*), hemolysin expression‐modulating proteins, and Type III hemolysin [70].

**Figure 3:**
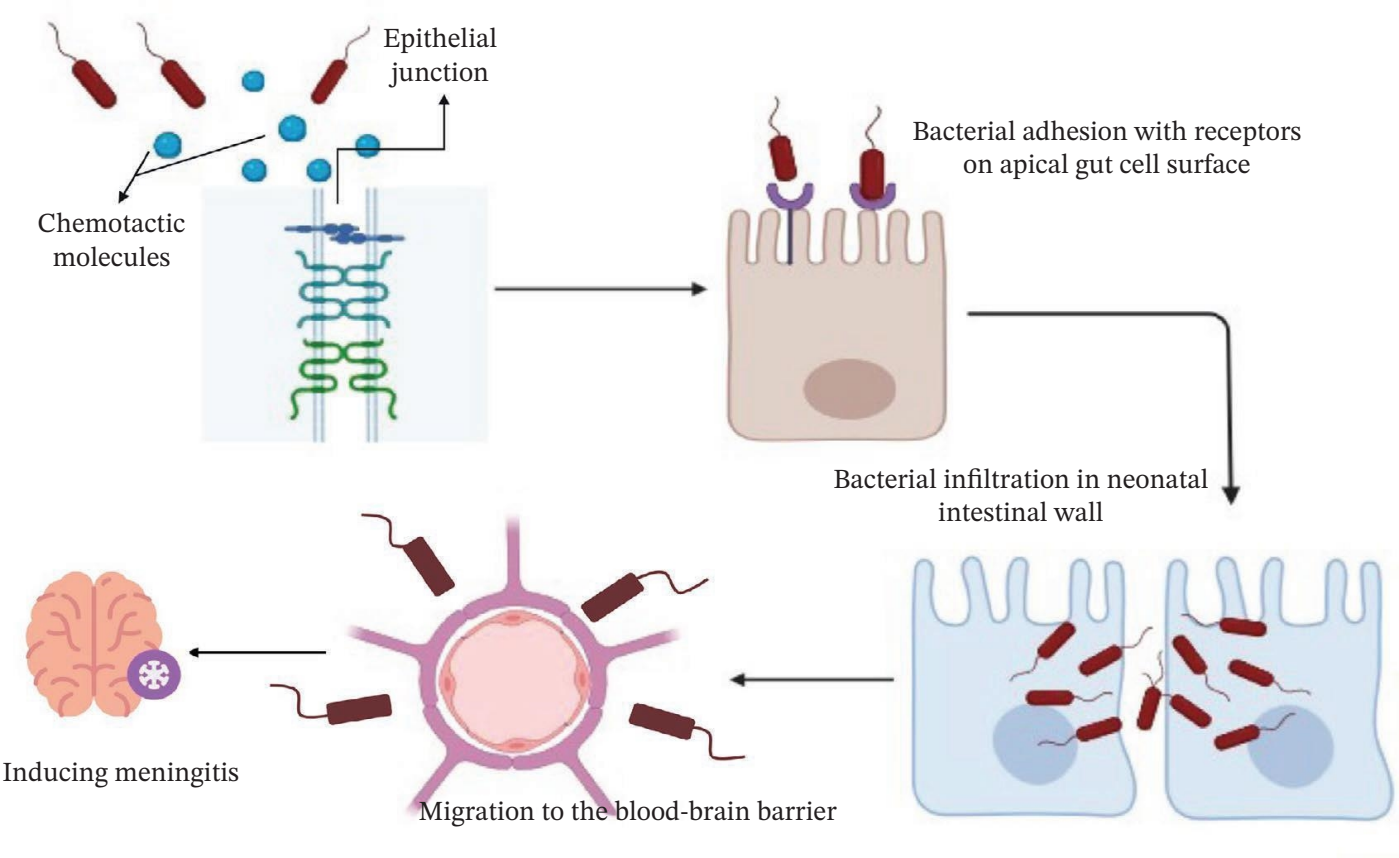
Pathogenesis of *Cronobacter sakazakii* causing neonatal meningitis. Figure generated by http://biorender.com/.

After invading intestinal epithelial cells, bacteria replicate intracellularly. Subsequently, the bacteria migrate to the apical surface, undergo transcytosis into the lamina propria, and utilize macrophages and lymphoid cells for systemic dissemination. They subsequently induce meningitis once they infiltrate extraintestinal locations such as the blood–brain barrier [70]. The entire cycle of causing meningitis has been illustrated in Figure 3.

#### 3.5.3. Persistence Mechanisms in Host

AMR, iron acquisition, sialic acid consumption, and immune system invasion constitute a few of the persistence mechanisms that the bacteria implement [7], [70]. According to studies, strains with functioning RpoS are more resistant to oxidative, osmotic, and acidic stressors [75]. The overexpression of ibpA and ibpB genes after heat stress has been validated by proteomic analysis, highlighting their function in thermal resistance [75]. *ClpK* has been found to play a crucial role in mediating lethal heat shock tolerance in *C. sakazakii*, which improves the bacterium′s ability to survive in hot conditions [75]. One of the vital microelements that the illness‐triggering bacteria demand is iron. In *Cronobacter* spp., two iron acquisition mechanisms have been studied: ABC transport‐mediated iron uptake and iron acquisition system mediated by siderophore (*iucABCD* and *iutA*). In order to promote the proliferation of bacteria in human hosts, the *iucABCD* gene cluster contributed to the encoding of a siderophore‐mediated iron acquisition mechanism [70]. The genes encoding ferric enterobactin transport proteins have been identified as *entA*, *fepC*, *fepG*, and *entH* [7]. The proteins referred to as outer membrane proteins (*OmpC* and *OmpA*) promote the synthesis of energy along with the flow of inorganic ions [49].

#### 3.5.4. Evasion of Host Defense

The *nanAKT* gene cluster encodes enzymes that participate in sialic acid metabolism, particularly the catabolism of N‐acetylneuraminic acid, enabling bacteria to utilize host‐derived sialic acids as carbon sources. This metabolic adaptation has been associated with increased virulence in many infections by facilitating mucosal colonization and evasion of complement‐mediated destruction [50]. The genes, which permit bacteria to thrive in macrophages, are putative sod genes which also have an impact on bacterial survival in an acidic environment [76, 77]. The FKBP‐type peptidyl‐prolyl cis‐trans isomerase gene, *fkpA*, potentiates macrophage infectivity and is thought to be a virulence factor in *Cronobacter* spp. Within the macrophages that the host immune system secretes, these genes allow the cell to endure, proliferate, and live [52, 78]. The *sod* (superoxide dismutase) gene was also discovered in the genomic sequence and was correlated with *C. sakazakii*′s persistence in macrophages. This gene has been implicated in the production of hemolytic activity in bacterial cells [51]. The proteins *znuB* and *znuC*, assisting in ensuring bacterial survival in macrophages, have been designated as zinc ABC transporters, ATP‐binding proteins, and permease proteins and, correspondingly [7, 71].

### 3.6. Antimicrobial Susceptibility

Studies discovered 39 genes, *cpxA*, *CRP*, *emrAacrR*, *AcrZ*, *MarA*, *MarB*, *marR*, *FosA*, *GlpT*, *AcrAB-TolC*: *acrA*, *acrB*, *AcrAD-TolC*: *acrD*, *AcrEF-TolC*: *acrF*, *MdtABC-TolC*, *baeR*, *BcrC*, *SugE*, *qacE*, *EmrAB-TolC*: *emrB*, *emrR*, *gyrA* (*DNA-targeted antibiotic resistance*), *H-NS*, *MacA*, *yojI*, *sul1*, *mdfA*, *MdfA*/*Cmr*, *mdtA*, *mdtB*, *mdtH*, *QacE*, *tetA*, *tetC*, *and blaTEM-1* responsible for developing AMR in *Cronobacter* spp. [6, 7, 53, 54]. Proton motive forces drive RND‐type tripartite efflux pumps, such as AcrAB‐TolC, AcrAD‐TolC, AcrEF‐TolC, and MdtABC‐TolC pumps, which use various adaptors and transporters [53]. Small multidrug‐resistance (SMR) transporters, or *mdt* variants, are frequently a component of the BaeSR‐regulated mdtABCD operon [79]. Often discovered on integrons or plasmids, *SugE* and *qacE* encode genes of SMR proteins that provide resistance to disinfectants like benzalkonium chloride [55]. Regulatory and signal transduction pathways involve *H-NS*, *CRP*, *BaeR*, and *CpxA* [55–58]. *marA* and *AcrZ* genes, linked to transcriptional activators and multidrug efflux pumps, correspondingly, were present in isolates. The genes *blaTEM-1*, *aadA*, *BcrC*, *marR*, *marB*, *sul1*, *FosA*, and *GlpT* are involved in the antibiotic target alteration and inactivation [59].

The *C. sakazakii* found from PIF and RTE food in different countries and different regions of the world showed diverse results for their antimicrobial susceptibility and resistance patterns based on the minimal inhibitory concentration (MIC) values of the antibiotics, the time period of those studies and sometimes also based on the fact where the studies had been taken in account of. Antimicrobial sensitivity tests showed that all of the examined isolates were susceptible to ciprofloxacin, tetracycline, cefotaxime, and nalidixic acid, whereas 84.5% were resistant to penicillin G and 46.5% were resistant to cephalothin. These findings are reported in Xu et al., 2015, a study conducted in China in 2015. A total of 280 samples were tested for the presence of *C. sakazakii*, and 18.6% of the samples tested positive for the organism [22]. According to Fei, Yichao Jiang, et al., 2017, this was conducted also in China with a higher number of samples, of which 2.8% were positive for the organism. All of the antibiotics, such as ampicillin sulbactam, ciprofloxacin, meropenem, cefotaxime, piperacillin‐tazobactam, tetracycline, and trimethoprim‐sulfamethoxazole, were effective against the *C. sakazakii* isolates. Furthermore, it was shown that whereas the majority of those isolates (92.9% and 87.5%, respectively) were sensitive to gentamicin and chloramphenicol, 55.4% of the *C. sakazakii* strains were cephalothin resistant [5].

*Cronobacter* strains were susceptible to the majority of the antibiotics, including amikacin, aztreonam, cefepime, ampicillin–sulbactam, chloramphenicol, cefotaxime, ceftazidime, ciprofloxacin, gentamicin, colistin, imipenem, meropenem, moxifloxacin, levofloxacin, piperacillin‐tazobactam, piperacillin, tetracycline, and trimethoprim‐sulfamethoxazole, according to Fei, Yujun Jiang, et al. (2017), but they were ampicillin, amoxicillin–clavulanate, and cefazolin resistant [5]. A different study led in 2020 revealed that of 72 isolates in total, 68 isolates (94.4%) were resistant to cefazolin, 7 isolates (9.45%) to amoxicillin, 4 isolates (5.55%) to cefpodoxime, 1 isolate (1.35%) to trimethoprim–sulfamethoxazole, and 1 isolate (1.35%) to streptomycin. Furthermore, two isolates were simultaneously resistant to those three drugs [60]. Another study was conducted in 2021, and it suggested that all *Cronobacter* strains isolated in the study were sensitive to trimethoprim/sulfamethoxazole, gentamicin, and chloramphenicol. Cephalothin (85.2%) was the most commonly observed form of resistance, followed by cefoxitin (33.3%), cefotaxime (14.8%), and ampicillin (11.1%) [61]. Out of the 27 *Cronobacter* isolates from 3600 samples, the majority of the isolates in Fei, Jing, et al.′s investigation (2022) demonstrated resistance to vancomycin and cephalothin, demonstrating a similar level of resistance to most antibiotics as in previous studies; however, all of the isolates were susceptible to all sulfonamide antibiotics [10]. According to the study, all isolates were shown to be susceptible to ceftriaxone, cefotaxime, gentamicin, colistin, ampicillin–sulbactam, sulfadiazine, sulfadoxine, and trimethoprim‐sulfamethoxazole. The bulk of isolates were revealed to be susceptible to the following antibiotics: ampicillin (81.48%), oxytetracycline (96.30%), pipemidic acid (96.30%), erythromycin (88.89%), streptomycin (88.89%), amoxicillin (85.19%), and azithromycin (92.59%). Meanwhile, 55.56% and 96.30%, respectively, of the strains were resistant to vancomycin and cephalothin [10]. In a separate investigation, their team discovered that all 32 strains of *C. sakazakii* isolated from 750 goat milk–based formula (GIF) were susceptible to imipenem, aztreonam, ampicillin/sulbactam, and trimethoprim–sulfamethoxazole. The isolates had resistance rates of 91.91% on average to cephalothin, and 3.68% of them were intermediate, respectively. CroM234A1 and croM283‐1, two of the identified strains, showed resistance to three different classes of antibiotics [10]. After this, another article was published in China in late 2022, where 96 different strains of *Cronobacter* were tested to see how well commonly used antibiotics in hospitals could inhibit or kill them. This was done using standards from the Clinical and Laboratory Standards Institute (CLSI). The isolated 96 *Cronobacter* strains in that study were completely resistant to macrolide antibiotics. Of all cephalosporin antibiotics tested, the *Cronobacter* strains responded most strongly to ceftazidime (88.7%) and less strongly to cefazolin (34.0%). The organisms were found more resistant to erythromycin and midecamycin compared with vancomycin and clindamycin. *Cronobacter* showed high resistance to neomycin (68.0%) and amikacin (54.6%), whereas resistance to gentamicin (20.6%) and kanamycin (24.7%) was much lower. The organism was more susceptible to penicillin antibiotics than other antibiotics. Its susceptibility to penicillin antibiotics was about 50%. Research found *Cronobacter* to be more easily inhibited by tetracycline and doxycycline, but ofloxacin and norfloxacin could not destroy them [62]. All these studies suggest that the resistance pattern of the organism changes over the period of the studies and also within the geographical location of the samples or, more specifically, country‐wise. Notably, ampicillin resistance was consistently high in Asia compared with other continents. Besides, over the years, an alarming increase rate of gentamicin resistance in China has been observed along with frequent reports of chloramphenicol resistance [5, 10, 16, 22, 62]. In contrast, ampicillin–sulbactam showed good activity against these isolates. However, this antibiotic was found to be ineffective against isolates from Chile and Iran [62, 63]. Again, in South America, *Cronobacter* isolates were found to be resistant to cefotaxime, ceftazidime, and cephalothin more than any other region [6, 64]. However, we have to keep in mind that variations in reported contamination rates may indicate international discrepancies in antibiotic stewardship and manufacturing processes for PIF, encompassing disparities in sterilization methods, facility hygiene standards, and regulatory supervision.

### 3.7. Sign and Symptoms

**Figure 4:**
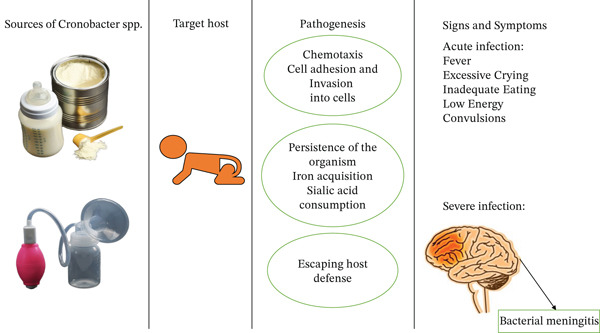
Source, pathogenesis, and sign‐symptoms of *Cronobacter sakazakii* infections.

Although rare, *Cronobacter* infections can be rather serious, particularly in infants under 1 year of age. The following are indications and manifestations linked to infections with *C. sakazakii*: (1) Infants (under a year old): Babies can exhibit a variety of symptoms, including low energy, excessive crying, poor eating, fever, and seizures in certain cases. *Cronobacter* bacteria can enter the bloodstream of infants, particularly those under 2 months old, and cause meningitis, which is an inflammation of the membranes that surround the brain and spine. Meningitis babies may experience severe, lifelong brain abnormalities. In the United States, 20% of newborns with bloodstream infections or meningitis caused by *Cronobacter* spp. have died, whereas 40% of instances recorded globally have resulted in death [25]. (2) People of all ages: Older children and adults are also susceptible to infections caused by *Cronobacter*. Septicemia, or difficulties in cuts, scrapes, or surgical sites, can result from it. UTI infections can also be caused by *Cronobacter*. Infections by *Cronobacter* spp. are more common in those 65 years of age and older, as well as in people whose immune systems have been compromised by disease, immunosuppressive medications, HIV infection, or genetic disorders [25]. Sources, pathogenesis and signs and symptoms of the diseases caused by *C. sakazakii* have been shown in Figure 4.

### 3.8. Management and Control

#### 3.8.1. Prevention

There are overall three measures to stop *Cronobacter* spp. from spreading and causing infections in infants and children. The first of them is associated with the packaging of PIF and other RTE food for babies. The first stage where the food can actually be contaminated with *Cronobacter* spp. is the production of formula foods, and the most important source after the production worker is the packaging material of those foods. Proper maintenance of hygiene for the production worker is so important, and what else is important here is maintaining a level of packaging where there is no chance of the packaged food being contaminated with *Cronobacter* spp. One study focused on the inhibitory effect of silver nanoparticles/polymethylmethacrylate/cellulose acetate (AgNP/PMMA/CA) films on *C. sakazakii* in infant formula milk. The film showed antibacterial activity and can potentially be used as a packaging material or as an antibacterial surface coating on packages for PIF and RTE foods [20]. The second and third stages, where necessary steps may prevent the *Cronobacter* infections, rely on the preparation of the food before consumption. Another study was conducted back in 2015 based on the inactivation of *C. sakazakii* in PIF which suggested that thymoquinone (TQ) and mild heat might be used in infant formula to control *C. sakazakii* before consumption, which could be a potential means of preventing infections linked to *C. sakazakii* in infant formula [65]. There is another study focused on the use of triglycerol monolaurate (TGML) that demonstrated that TGML maintained a high level of consumer sensory acceptance while demonstrating a strong inhibitory action on *C. sakazakii.* Their bacteriostatic activity was increased by lowering the pH and/or adjusting the temperature [66]. When compared with either therapy alone, the combination of 405‐nm LED light and citral offers better antibacterial effects in both PBS and reconstituted PIF. Moreover, *C. sakazakii*′s cell membrane shape is disrupted, its membrane integrity is weakened, and the recovery time of sublethally wounded bacteria is lengthened when 405 nm LED and citral therapy are combined. Furthermore, *C. sakazakii*′s lipid peroxidation is decreased by LED therapy [19]. Some other studies as well as the CDC suggest that properly cleaning and sanitizing breast pump equipment for feeding babies plays a crucial role in preventing such infections. In addition, proper maintenance of good hygiene practices and following guidelines are necessary for preventing any bacterial infections, including *C. sakazakii* [39]. There are three currently available methods for decontaminating the PIF and RTE production area and the equipment used to feed children: (i) use of disinfectants to eliminate biofilm attachment from biotic surfaces, infant formula preparation areas, laboratories and hospitals, food services, and child day care settings; (ii) thermal inactivation for both biotic and abiotic surfaces, and (iii) high‐pressure processing (HPP) or high hydrostatic pressure (HHP) for nonthermal processing in closed water‐filled chambers [39]. There should be necessary concerns to prevent the severity of *Cronobacter* infection if one already becomes infected. The need for wider whole‐genome sequencing (WGS) implementation as part of the diagnostic procedure is very important. Early detection and appropriate management are crucial for preventing severe infections caused by *C. sakazakii* [67]. Aptamers could also be used to detect *Cronobacter* with ease to detect the organism.

#### 3.8.2. Diagnosis

Currently, there are a number of techniques for identifying the bacterium that triggers the disease, and efforts are underway to establish innovative methods for more rapid and straightforward detection. The customary approach ISO/TS 22964:2017 for identifying *C. sakazakii* in PIF stands on a culture protocol for initial isolation and biochemical appraisal for final verification. Because of the particular requirements for cultivation conditions and reagents, this procedure takes 5–7 days and demands a lot of effort [68, 80]. As last stages to replace the biochemical testing, additional approaches including polymerase chain reaction (PCR) [81], immunosorbent assays linking with enzyme [31], or assays dependent on immunoliposome [32–34] have been proposed as potential substitutes for culture‐based approaches in the detection of *C. sakazakii* as per the “The customary approach defined in ISO/TS22964:2017 for identifying *C. sakazakii* in PIF [35]. Although less laborious, molecular techniques for identifying *C. sakazakii* need the creation of certain primers and specialized tools. Only the *rpoA* together with 16S rRNA gene sequences were thought to may be used to describe between *C. sakazakii* and *C. malonaticus* [36]. However, due to their significant genetic similarity, *C. sakazakii* and *C. malonaticus* cannot be reliably differentiated just by 16S rRNA or *rpoA* gene sequences. *FusA* sequencing offers a rapid method of identifying the species and it offers more reliable data than 16S rRNA for species differentiation, although requiring 24–48 h to run the sequencing [36]. PCR‐based serotyping was thought to be a practical technique for quickly and precisely typing a variety of *Cronobacter* species. However, along with PCR analysis, the complete *Cronobacter* genus has been reported to be assigned in a MLST to analyze organizational variations across seven housekeeping genes (namely, *atpD*, *fusA*, *gltB*, *gyrB*, *infB*, *ppsA*, *rpoB*) for accurate species identification [30]. It takes 2–3 days for the whole processing to get the results and costs a lot but their accuracy level is high enough to rely them for epidemiological analysis [30, 82]. Until May 2025, the PubMLST database contains over 4000 *Cronobacter* strains with more than 1700 distinct STs. Among the 3015 *C*. *sakazakii* isolates in the *Cronobacter* PubMLST database (until May 2025), clonal complex 4 (CC4) is the most prevalent lineage associated with neonatal meningitis, accounting for about 25% of the strains (https://pubmlst.org/Cronobacter/). Although CC4 constitutes over 80% of meningitis cases, CC1 (8% of isolates) and CC8 (3% of isolates) have been intermittently linked to newborn infections, albeit with reduced clinical frequency [25]. Of the 3015 isolates of *C. sakazakii* identified in the MLST database, is persistent alongside predominantly linked to neonatal meningitis. Additionally, the Clusters of Orthologous Groups–core genome (COG–cg) MLST (1865 locations) and the ribosomal‐MLST (53 loci) have confirmed CC4 as the primary lineage [28]. Enzyme‐linked immunosorbent assays (ELISA) [31] have been proposed as the final endorsement stages to replace the biochemical testing [35]. The applications of highly specific aptamers, which are essentially single‐stranded, short DNA (ssDNA) or RNA oligonucleotides generated in vitro, as bio‐receptors alongside detection tools [83, 84] offer a feasible alternative to conventional techniques of identifying pathogens. Centrifugation‐based partitioning (CBPM) and aptamer extraction frequently employs whole‐cell systematic evolution of ligands by exponential enrichment (SELEX) [85, 86]. According to the Raman signal engendered by the gold nanoparticles′ p‐aminothiophenol interaction, a precise and quantitative test technique was created. The linearity range of the SERS immunochromatographic (IC) test strip assay was 10^2^–10^7^ cfu/mL, and it only took 12 min to analyze [87]. Furthermore, the LAMP‐LFD approach demonstrates significant importance and promise for quick identification of *C. sakazakii* in PIF [88]. Furthermore, because of antibodies against *C. sakazakii* being commercially inaccessible, they must be generated internally, which adds to the technical hurdles of assays based on immune molecules, which rely on antibody–antigen interconnection [64, 89, 90]. Thus, it is urgently necessary to establish straightforward, quick, and inexpensive approaches for detecting *C. sakazakii*–specific receptors.

##### 3.8.2.1. Molecular Diagnosis System Based on Labchip

The molecular steps are a simple, cost‐effective, streamlined, and intuitive way of diagnosing the organism including improving the PIF sample, mixing the sample using the MasterMix kit, loading the labchip straight into the mixture, and inserting the labchip into the rapid thermal cycler [91]. This approach can help prevent misleading results attributed to the experimenter’s insufficient proficiency with detection technologies (beyond standard culture testing) as it requires no specialized personnel. It renders detection more straightforward and reliable through a diminution of detection costs, the simplicity of analytical processes, and the precise and swift regulation of reaction temperature, including the shrinking of detection equipment. In this case, inserting the enriched sample homogenate right away elevates usability instead of necessitating postenrichment. The recognition of this novel detection strategy might be as low as the lowest concentration of the inoculum that is available for growth after 12 h of enrichment with DW (about 1 cfu/300 g PIF). Biodegradable labchips are manufactured employing plastic to alleviate expenses and increase heat exchange precision by accurately controlling chip thickness in response to the temperature of response. This heating method′s rate of fluctuations in temperature was 20°C/s–40°C/s. Overall, this approach supports the on‐site examination and timely remedial measures [91].

##### 3.8.2.2. Adapter‐Based Detection Methods

###### 3.8.2.2.1. Aptasensor

The aptamer docking region, the G‐quadruplex complementary region, and the double‐strand stem area comprised the three sections of the hairpin DNA intended for the RCA template. Two hairpin DNA molecules might generate a circular, dumbbell‐shaped DNA template as an RCA template in the presence of T_4_ DNA ligase; this method is simpler to build and less expensive than alternative approaches. Following centrifugation, the remaining aptamers from the bacterial binding of the samples are utilized in the rolling circle amplification (RCA) test. Attached to a template in the RCA assay, aptamers launch RCA and produce G‐quadruplex structures. ThT, a fluorescent dye, enhances fluorescence by intercalating into these frameworks. Aptamers stay in suspension and demonstrate more fluorescence in the RCA test when the bacteria are not extant. A fluorescence readout device may be used to monitor the fluorescent intensity, which is correlated with the target bacteria′s abundance [92]. The suggested aptasensor may be used at a constant temperature (37°C) for the duration of the detection process and does not require complex antibodies, heat cycle equipment, or DNA extraction stages. To lessen the impact of the PIF matrix, the suggested assay can also be used in conjunction with pre‐enrichment techniques such as the magnetic separation system. According to the findings, the aptasensor demonstrated high specificity for *C. sakazakii* detection in both inclusiveness and limitation tests. Additionally, after 3 h, the aptasensor could identify *C. sakazakii* at concentrations 10^2^ cfu/mL in pure culture, down to as little as 10^3^ cfu/mL in PIF samples. In pure culture, the suggested aptasensor recovered between 104% and 111%, whereas in PIF samples, it recovered between 96% and 107%. These findings suggested that there was a lot of promise for the aptasensor suggested here in terms of identifying *C. sakazakii* [92]. In under 30 min, Kim et al. reported on a label‐free aptasensing platform that used gold nanoparticles to detect *C. sakazakii* in PIF with a sensitivity of 7.1 × 10^3^ cfu/mL [93].

###### 3.8.2.2.2. ssDNA Aptamer

A CBPM for screening aptamers with specificity and high affinity for bacteria eliminates the need for the repeated enrichment step in order to elute DNA, making it quicker than SELEX (systematic evolution of ligands by exponential enrichment) [86, 93]. Cells of *C. sakazakii* were cultured using a random DNA library pool. The DNA that was firmly attached to the cells was retrieved following the last partitioning, which involved the successive removal of unattached or incompletely bound DNAs via CBPM. The approach shortens the time needed for analysis and makes it possible to identify *C. sakazakii* contamination in food with accuracy. Other *Cronobacter* species can be isolated using the aptamer isolating technique (CBPM) to generate target‐specific probes that can be utilized in multiplex detection systems to identify other *Cronobacter* species. The aptamers generated in this investigation, however, showed cross‐reactivity in their sensing platform with additional species of *Cronobacter*, including *C. turicensis*, *C. condimenti*, *C. muytjensii*, *C. dublinensis*, *C. universalis*, *or C. malonaticus* [86].

##### 3.8.2.3. Bioluminescent Reporter Phage

A bioluminescent reporter phage, *Φ*C01_lux_*Δ*hol, was developed as a rapid and specific tool to detect live *C. sakazakii* cells. This phage was engineered by integrating the *luxCDABE* operon and modifying the holin protein to delay bacterial lysis, allowing for a more consistent and prolonged bioluminescent signal. Using this method, even very low levels of *C. sakazakii* as few as 2 cfu/mL could be detected in PIF and on food‐contact surfaces within just 7 h, including a 5‐h enrichment period [29].

##### 3.8.2.4. Immunochromatography

Methods based on IC strips have become useful instruments for *C. sakazakii* detection because they provide a compromise between sensitivity, speed, and usability. After a 6‐h enrichment and a further 2.5‐h assay period, Cho et al. created an immunoliposome‐based IC assay combined with immunomagnetic nanoparticle concentration that can detect as little as two cells/10 g of PIF [34]. Without being impacted by food matrix interference, some techniques surpassed RT‐PCR and ELISA and showed greater specificity. With a limit of detection (LoD) of less than 10 cells, Blazkova et al. presented an ultra‐rapid IC strip that targets the 16S rRNA gene and uses PCR amplicons labeled with digoxigenin and biotin [94]. This allowed for visual detection in less than 10 min. This approach is especially well‐suited for low‐resource environments because it requires very little equipment. Using silica‐coated magnetic nanoparticles and hybridization with 16S rRNA probes in a gold nanoparticle‐based IC format, Yan et al. achieved clear red‐line results with a LoD of 10^6^ cfu/mL, which are appropriate for direct testing in PIF samples [94]. When taken as a whole, these IC platforms show a variety of solutions designed to meet different field and laboratory requirements, ranging from high sensitivity to quick screening.

**Figure 5:**
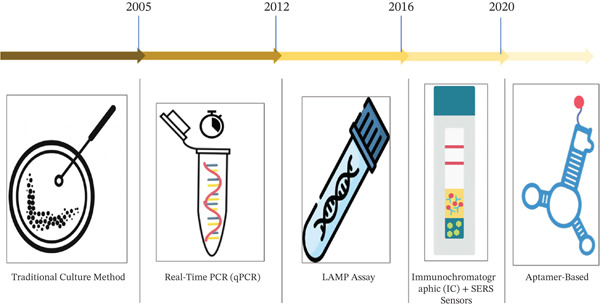
Development of different detection methods of *Cronobacter sakazakii*.

By illustrating the chronological development of detection methods in Figure 5, a comparison among the above‐mentioned methods has been shown in Table 3 based on their total time to give result, LoD, specificity, sensitivity, and cost.

**Table 3:** Comparison among the detection methods of *Cronobacter sakazakii*.

Detection method	Duration (pre‐enrichment)	Limit of detection (LoD)	Detection type	Specificity and sensitivity	Cost/field applicability	Ref.
LabChip RT‐PCR (2020)	30 min assay (12 h)	1 CFU/300 g PIF (~10.4 genome copies)	DNA (RT‐PCR)	High: 100% sensitivity and specificity (n = 50/50)	Low cost; portable; minimal training required	[91]
Bioluminescence based techniques (2021)	7 h (including 5 h for incubation)	Very low levels as few as 2 CFU/mL	Bioluminescent signal	Highly specific	Moderate; portable luminometer required	[28]
RCA‐based Aptasensor (2021)	~3 h (centrifugation step only)	10^2^ CFU/mL (pure culture), 10^3^ CFU/mL (PIF)	Viable cells via aptamer‐RCA	High specificity; 96%–111% recovery; no DNA extraction needed	Low cost; isothermal; no complex instruments; field‐suitable	[92]
Label‐free AuNP aptasensor (2017)	< 30 min	7.1 ∗ 10^3^ CFU/mL (PIF)	Whole cells via aptamer–gold interaction	High specificity; rapid visual detection	Very low cost; equipment‐free; excellent for field use	[93]
IC + immunoliposome + magnetic separation	2.5 h (6 h)	2 cells/10 g PIF	Whole cells	High specificity; better than RT‐PCR and ELISA; matrix independent	Moderate; requires lab prep of liposomes and magnetic particles	[31]
IC strip + PCR amplicon	10 min (16 h)	< 10 cells (~8 ng DNA)	16S rRNA gene	High specificity; visually distinguishable results	High field suitability; minimal instrumentation required	[95]
IC strip + magnetic NP+16S rRNA probe	2–4 h	10 CFU/mL	16S rRNA gene (hybridized probe)	High specificity; balanced speed/performance	Good for field use; easy visual readout	[96]
Conventional culture (ISO/TS 22964:2017)	5–7 days	Differs; typically 10 CFU/25 g	Viable cells	High specificity; time‐consuming and labor‐intensive	Low cost but not field‐suitable; requires lab setup	[66]
FusA Gene Sequencing	~24–48 h	Not CFU‐based; DNA‐level identification	fusA gene (species‐level)	More reliable than 16S rRNA for species differentiation	Moderate; lab setting with sequencing capability needed	[33]
MLST (multilocus sequence typing)	2–3 days	Not applicable (typing method)	7 housekeeping genes	High accuracy for strain‐level differentiation	High cost; suitable for epidemiology, not routine screening	[28, 35]
Advanced SERS with gold NP (2020)	12 min	10^2^–10^7^ CFU/mL	Whole cell Raman signal	Precise, quantitative, and rapid	High instrumentation cost; lab‐based	[86]
LAMP‐LFD (lateral flow device)	30–60 min (optional)	~10^2^ CFU/mL	DNA amplification (LAMP)	High sensitivity and rapid visual output	Low cost; portable and field‐friendly	[87]

##### 3.8.2.5. Artificial Intelligence (AI)–Based Detection

In recent times, detection of foodborne pathogens has been significantly improved using AI‐based biosensors [96]. Although biosensors alone can detect foodborne pathogens fast, AI enhances their real‐world reliability by reducing signal noise, handling sensitivity variability, and overcoming interference from complex food matrices [95]. In 2021, Raman spectroscopy combined with ML has rapidly identified 23 common strains from seven genera, including *C. sakazakii*, at single‐cell level with a very high accuracy rate of 86.23*%* ± 0.92*%* [97]. However, there were high errors in case of certain strains. Later, incorporation of self‐transfer deep learning and ensemble prediction algorithms has increased the accuracy rate to more than 99% [98]. Deep learning strategies enhance biosensor capabilities by automatically extracting hierarchical features from raw data, making them specifically suitable for large‐scale and high‐dimensional datasets. Deep learning models provide high adaptability and generalization, which strengthens their robustness and reliability across a wide range of biosensor platforms [95, 99].

#### 3.8.3. Antibiotic Intervention

Much like the other bacterial infections, the choice of treatment for *Cronobacter* infection vastly relies on antibiotic treatments. This is a rare bacterium found only in some specific sources and studies suggest that *Cronobacter* spp. are still not that much resistant to most of the antibiotics like the common pathogenic bacteria [5, 16, 22, 40, 61] suggest that *C. sakazakii* is 100% susceptible to ampicillin–sulbactam, meropenem, tetracycline, piperacillin–tazobactam, cefotaxime, ciprofloxacin, and trimethoprim–sulfamethoxazole. The organism has also been found to be mostly susceptible to chloramphenicol and gentamicin as well, but not all [5], [22]. In some other cases, ampicillin, amoxicillin, and ciprofloxacin were found to be resistant to *C. sakazakii* [63, 100]. A complete clinical examination, including blood, urine, and cerebrospinal fluid cultures, should be provided to infants suspected of having meningitis or sepsis upon hospital admission, according to recommendations made by the CDC. Patients should receive empirical antibiotics as soon as *Cronobacter* is detected. The existence of multidrug‐resistant microorganisms makes antimicrobial susceptibility testing necessary. Additionally, some research demonstrated that in the LIVE/DEAD experiment, LED‐treated bacteria might harm the bacterial cell membrane [18]. Once more, the antibacterial effects of citral and 405‐nm LED illumination were superior to those of either therapy alone [19]. The use of AgNP/PMMA/CA (silver nanoparticles/polymethylmethacrylate/cellulose acetate) films is an additional strategy for preventing *C. sakazakii* in infant formula [20].

## 4. Future Outlook

### 4.1. The Role of Molecular and AI‐Based Technologies in *C. sakazakii* Control and Management

The future of *C. sakazakii* detection and control is poised for a transformative shift, moving away from traditional, time‐consuming culture‐based methods towards a new paradigm driven by molecular diagnostics, genomics, and AI. The integration of these advanced technologies promises to deliver unprecedented speed, accuracy, and predictive capability in safeguarding public health, particularly for vulnerable infant populations.

#### 4.1.1. Advanced Molecular Diagnostics and Genomic Surveillance

The cornerstone of future detection will be the widespread adoption of rapid, on‐site molecular techniques. Although PCR is now standard, emerging isothermal amplification methods like loop‐mediated isothermal amplification (LAMP) and recombinase polymerase amplification (RPA) are set to become mainstream. These techniques can detect *C. sakazakii* with high specificity directly from complex matrices like PIF within a very short time without the need for sophisticated thermocycling equipment, enabling real‐time monitoring at production and point‐of‐use. In addition, the integration of CRISPR‐Cas systems with these amplification methods is creating next‐generation biosensors with single‐copy sensitivity and the ability to distinguish live from dead cells. WGS will transition from a research tool to a routine public health application. WGS provides the highest possible resolution for strain typing, enabling precise traceback of contamination sources during outbreaks and distinguishing true transmission events from sporadic cases. This allows for targeted and effective recall actions. As sequencing costs continue to fall, the establishment of global genomic databases for *C. sakazakii* will facilitate real‐time international surveillance and the early identification of emerging virulent or resistant clones.

#### 4.1.2. The Transformative Potential of AI and ML

AI and ML are expected to revolutionize *C. sakazakii* risk management in several key areas including predictive microbiology and risk assessment, genomic analysis and pathogenicity prediction, and optimization of detection and control strategies. ML models can integrate vast datasets—including environmental parameters such as temperature, humidity, processing conditions, ingredient sources, and historical contamination records—to predict the growth and survival probability of *C. sakazakii* in PIF and production environments. These predictive models can dynamically assess risk and identify critical control points with greater accuracy than static models, allowing for pre‐emptive interventions. AI algorithms can rapidly analyze WGS data to predict phenotypic traits such as antibiotic resistance, stress tolerance, and virulence potential. By learning from genomic and associated metadata, ML models can identify genetic markers linked to hypervirulence or persistence in dry environments, helping to prioritize the most hazardous strains for control. AI will enhance the design of molecular assays by optimizing primer/probe sequences for broader strain coverage. In processing facilities, AI‐powered computer vision systems integrated with sensors can monitor hygiene in real‐time, identifying potential biofilm formation or procedural breaches. Furthermore, AI can drive the development of smart sanitation systems that autonomously adjust cleaning protocols based on real‐time microbial load data. The most significant advancement will come from the synergy of these technologies. Imagine a future where a portable LAMP‐CRISPR device detects *C. sakazakii* at a factory, instantly uploading the genomic data to a cloud‐based platform. An AI system then analyzes the sequence, predicts its resistance profile and persistence capabilities, cross‐references it with global databases to identify the source, and automatically updates the risk model for the production line—all within hours. This integrated, data‐driven approach will shift the paradigm from reactive containment to proactive, precision prevention. We have prepared a summary in Table S1. In conclusion, the fusion of field‐deployable molecular diagnostics, comprehensive genomic sequencing, and powerful AI analytics holds the key to effectively mitigating the threat of *C. sakazakii.* The successful implementation of this technological framework will be crucial for ensuring the safety of PIF and other high‐risk foods, ultimately protecting our most vulnerable consumers.

## 5. Conclusions

*C. sakazakii* represents a significant global public health concern, particularly for neonates and immunocompromised infants consuming PIF. Its ability to persist under harsh environmental conditions, form biofilms, and harbor virulence and AMR determinants underscores the complexity of its control. Although most strains remain susceptible to several frontline antibiotics, the emergence of resistant isolates highlights the need for ongoing surveillance and standardized antimicrobial testing. Preventive measures, ranging from stringent manufacturing practices and effective decontamination strategies to caregiver awareness of safe formula preparation, remain central to reducing infection risk. Advancements in rapid, cost‐effective, and reliable detection methods, alongside the exploration of novel interventions such as LED‐based treatments and antimicrobial packaging, hold promise for strengthening food safety. Ultimately, coordinated international efforts integrating research, regulatory oversight, and industry practices are critical to ensuring the microbiological safety of PIF and safeguarding infant health worldwide.

**Abbreviations**
AgNP: Silver nanoparticles
AMR: Antimicrobial resistance
BaeSR: Two‐component regulatory system BaeS/BaeR
BBB: Blood–brain barrier
CA: Colanic acid
CC: Clonal complex
CDC: Centers for Disease Control and Prevention
CFU: Colony‐forming unit
CHROMagar: CHROMagar E. sakazakii (chromogenic agar for selective isolation).
CM: Brilliance Chromogenic Agar CM 1035
CLSI: Clinical and Laboratory Standards Institute
CPS: Capsular polysaccharides
CRP: cAMP receptor protein
CSF: Cerebrospinal fluid
DFI: Druggan–Forsythe–Iversen agar
DNA: Deoxyribonucleic acid
DW: Distilled water
ELISA: Enzyme‐linked immunosorbent assay
EPS: Extracellular polymeric substances
FDA: Food and Drug Administration
GC: Guanine–cytosine content
HHP: High hydrostatic pressure
HPP: High‐pressure processing
IC: Immunochromatographic
ISO/TS: International Organization for Standardization—Technical Specification
LED: Light‐emitting diode
LFD: Lateral flow dipstick
LPS: Lipopolysaccharide
LAMP: Loop‐mediated isothermal amplification
LoD: Limit of detection
MLST: Multilocus sequence typing
mLST‐Vm: Modified lauryl sulfate tryptose broth–vancomycin medium
PBS: Phosphate‐buffered saline
PCR: Polymerase chain reaction
PIF: Powdered infant formula
PMMA: Polymethylmethacrylate
PRISMA: Preferred Reporting Items for Systematic Reviews and Meta‐Analyses
PROSPERO: International prospective register of systematic reviews
RCA: Rolling circle amplification
RND: Resistance–nodulation–division efflux pumps
RTE: Ready‐to‐eat
RpoS: RNA polymerase sigma factor S
rRNA: Ribosomal RNA
RT‐PCR: Reverse transcription polymerase chain reaction
SERS: Surface‐enhanced Raman scattering
SELEX: Systematic evolution of ligands by exponential enrichment
SMR: Small multidrug resistance transporter
ST: Sequence type
TQ: Thymoquinone
TGML: Triglycerol monolaurate
UTI: Urinary tract infection
WHO: World Health Organization
FAO: Food and Agriculture Organization

## Author Contributions

Sutapa Bhowmik: writing—original draft, validation, methodology, investigation, data curation. Sangita Ahmed: writing—review and editing, validation, supervision, methodology. Fatema Hasan Kaifa and Md. Abdullah Safi Al Safi: Original draft, writing—review & editing, validation, resources, methodology. Supantha Rivu: original draft, writing—review & editing, validation, resources, methodology. Md. Latiful Bari: writing—review and editing, validation, methodology, conceptualization. Md. Aminul Islam: writing—review and editing, methodology. Nayan Chandra Mohanto: writing—review and editing, supervision, methodology, conceptualization. Sangita Ahmed, Fatema Hasan Kaifa, and Md. Abdullah Al Safi have contributed equally.

## Funding

No funding was received for this manuscript.

## Conflicts of Interest

The authors declare no conflicts of interest.

## Supporting information


**Supporting Information** Additional supporting information can be found online in the Supporting Information section. Table S1 provides the summary of future technologies including molecular diagnosis, genomic surveillance, AI and ML, and integrated system for *C. sakazakii* detection and management.

## Data Availability

Data sharing is not applicable to this article as no datasets were generated or analyzed during the current study.
