# Supplementary material for: From Powder to Pathogen: A Systematic Review of Detection, Pathogenicity, and Mitigation of Cronobacter sakazakii in Infant Formula
Source: Int J Microbiol. 2026 Jun 7;2026:7658756. doi: 10.1155/ijm/7658756 (PMC13243874; doi:10.1155/ijm/7658756)
Supplement: Supplementary file 1 — Supporting Information Additional supporting information can be found online in the Supporting Information section. Table S1 provides the summary of future technologies including molecular diagnosis, genomic surveillance, AI and ML, and integrated system for C. sakazakii detection and management. [file IJM-2026-7658756-s001.docx]

**Supplementary table 1: Future technologies for *C. sakazakii* detection and management**

| Technology category | Specific technology | Future application & role | Key benefit / impact |
| --- | --- | --- | --- |
| Molecular diagnostics | LAMP, RPA | Rapid, on-site detection directly in production facilities or points of use. | Drastically reduces detection time from days to <30 minutes; enables real-time decision-making. |
|  | CRISPR-Cas Biosensors | Highly specific and sensitive detection, often coupled with amplification, to distinguish viable cells. | Provides ultra-sensitive, field-deployable diagnostics and more accurate risk assessment. |
| Genomic surveillance | Whole-Genome Sequencing (WGS) | Routine use for outbreak investigation, source tracking, and routine surveillance of strains. | Enables high-resolution traceback to the precise source of contamination, preventing further spread. |
| AI & ML | Predictive Microbiology Models | Integrates multiple data sources (environment, process data) to forecast growth and survival risks. | Moves from reactive to proactive control by predicting hazards before they occur. |
|  | Genomic Analysis & Pathogenicity Prediction | Analyzes WGS data to predict antibiotic resistance, virulence, and persistence traits of isolates. | Identifies and prioritizes the most high-risk strains for targeted intervention. |
|  | AI-Optimized Control Systems | Powers smart sanitation and computer vision for real-time hygiene monitoring in factories. | Automates and optimizes control measures, improving efficiency and reducing human error. |
| Integrated systems | Synergistic Platform (e.g., LAMP + WGS + AI) | Combines rapid detection, genomic identification, and predictive analytics into a single workflow. | Creates a powerful, proactive framework for precision prevention and real-time risk management. |
